# Physicochemical Stability of Dentin-Derived Biomaterials During Long-Term Storage

**DOI:** 10.3390/jfb17060284

**Published:** 2026-06-07

**Authors:** Robert Dłucik, Alberto Scoglio, Domenico Puzzolo, Barbara Testagrossa, Angela Alibrandi, Antonio Toscano, Bogusława Orzechowska-Wylęgała, Giuseppe Acri

**Affiliations:** 1 Dłucik Dental Clinic, 40-737 Katowice, Poland; 2 Department of Biomedical and Dental Sciences and Morphofunctional Imaging, University of Messina, 98121 Messina, Italynico.puzzolo@unime.it (D.P.); barbara.testagrossa@unime.it (B.T.); giuseppe.acri@unime.it (G.A.); 3 IRCCS Centro Neurolesi “Bonino-Pulejo”, 98121 Messina, Italy; 4 Department of Human Pathology in Adult and Developmental Age “Gaetano Barresi”, University of Messina, 98121 Messina, Italy; angela.alibrandi@unime.it; 5 Department of Clinical and Experimental Medicine, University of Messina, 98121 Messina, Italy; antonio.toscano@unime.it; 6 Children’s Maxillofacial Surgery Clinic, Pediatric Surgery, Medical University of Silesia, 40-752 Katowice, Poland

**Keywords:** dentin-derived biomaterials, Raman spectroscopy, collagen–mineral interface, physicochemical stability, regenerative dentistry, tooth-derived grafts

## Abstract

Dentin-derived biomaterials are hierarchical collagen–mineral composites increasingly used as bio-based scaffolds for bone regeneration. However, the effect of prolonged storage of extracted teeth on their physicochemical integrity remains unclear. This study evaluated the stability of dentin-derived biomaterials following long-term refrigerated storage (up to six years) using Raman spectroscopy. Extracted human teeth were processed using three preparation systems (BonMaker, Tooth Transformer, and Smart Dentin Grinder), and Raman-derived indices describing mineral and collagen structure were compared with freshly extracted controls. No time-dependent changes were observed in mineral crystallinity, carbonate substitution, or collagen-related parameters, indicating preservation of the collagen–mineral interface during storage. In contrast, the observed differences were primarily associated with processing pathways. Tooth Transformer and Smart Dentin Grinder exhibited Raman profiles closely resembling native dentin, whereas BonMaker showed reduced mineral content and altered mineral–matrix balance consistent with its demineralization protocol. These findings demonstrate that dentin behaves as a structurally stable hierarchical composite, reflecting intrinsic structural organization that limits physicochemical degradation over time. Long-term storage does not compromise dentin integrity, supporting its use as a reliable source of biomaterial for regenerative applications and future tooth banking strategies.

## 1. Introduction

The performance of bio-derived scaffolds is strongly influenced by the integrity of their hierarchical organization, in which organic and inorganic phases are structurally integrated across multiple length scales [1,2,3,4,5]. In mineralized tissues, this organization is based on the interaction between type I collagen and carbonated hydroxyapatite nanocrystals, forming a hybrid composite responsible for mechanical stability, physicochemical integrity, and biological functionality [1,2,3]. Disruption of the collagen–mineral interface may compromise both structural organization and regenerative performance.

Dentin represents a highly organized collagen–mineral composite with structural characteristics closely resembling those of bone [6,7,8,9,10]. Due to its composition and biological origin, dentin has increasingly attracted interest as an autologous biomaterial for regenerative procedures in oral and maxillofacial surgery [11,12,13,14]. Previous studies demonstrated that dentin-derived grafts may provide favorable osteoconductive and structural properties while preserving components of the native extracellular matrix [15,16,17,18,19].

Recent advances in dentin-derived biomaterials have focused primarily on processing protocols that modify the relative contribution of mineral and organic phases [12,13,14,15,16,17,18]. Depending on the preparation strategy, dentin grafts may exhibit different physicochemical characteristics and collagen–mineral balance, potentially influencing their biological and structural behavior [15,16,17,18,20,21,22]. However, despite the increasing clinical use of dentin-derived biomaterials, limited information is available regarding their long-term physicochemical stability during storage.

Water-mediated interactions play a fundamental role in maintaining the structural integrity of mineralized collagenous tissues [9,10,23,24,25]. Both free and bound water contribute to collagen stabilization and interfacial coupling between the organic matrix and apatite crystals [9,10]. Preservation of these interactions may therefore be essential for maintaining the integrity of dentin-derived biomaterials during prolonged storage.

Raman spectroscopy provides a non-destructive and chemically sensitive method for simultaneous evaluation of mineral and organic components in dentin [26,27,28,29,30,31,32,33,34]. Raman-derived indices enable indirect assessment of mineral crystallinity, carbonate substitution, collagen organization, and mineral-to-matrix balance, making this technique particularly suitable for the characterization of hierarchical biomaterials.

While several studies have characterized the short-term physicochemical properties of dentin-derived materials immediately after preparation, the stability of these composites over multi-year storage periods remains largely unexplored. This study fills a critical gap by evaluating the physicochemical integrity of dentin scaffolds after six years of refrigerated storage, providing essential evidence for the long-term feasibility of tooth banking strategies.

Therefore, the aim of this study was to evaluate the physicochemical stability of dentin-derived biomaterials during long-term refrigerated storage using Raman spectroscopy. In particular, the study investigated whether prolonged storage influences the integrity of the collagen–mineral interface and whether the observed physicochemical characteristics are primarily associated with storage duration or processing-dependent modifications.

## 2. Materials and Methods

### 2.1. Study Design

**Figure 1:**
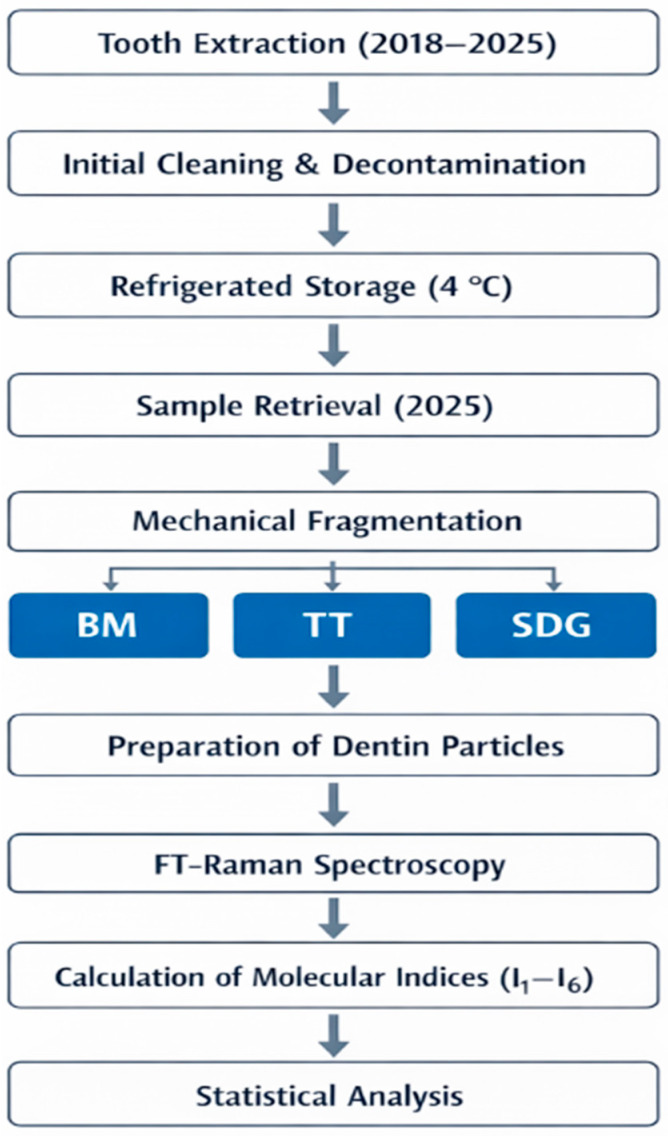
Schematic workflow of the experimental design.

This study evaluated the physicochemical stability of dentin-derived biomaterials following prolonged refrigerated storage using Raman spectroscopy. Extracted human teeth collected between 2018 and 2024 and stored under controlled conditions (4 °C) were used as the experimental material. Freshly extracted teeth obtained in 2025 served as controls representing native dentin without prior storage. The workflow is reported in Figure 1.

A total of 48 teeth extracted primarily for periodontal indications were included. Teeth were grouped according to the year of extraction (2018–2024), with six specimens per annual group (*n* = 6). Except for the control specimens, each annual group was subdivided according to the dentin-processing system used for biomaterial preparation: BonMaker (BM) (Korea Dental Solution Co. Ltd., Busan, Korea), Tooth Transformer (TT) (TT Tooth Transformer Srl, Milan, Italy), and Smart Dentin Grinder (SDG)( OMNIA Srl, Fidenza, Italy), representing chemically modified, mineral-dominant, and minimally processed approaches, respectively.

Sample preparation and initial processing were performed at Dłucik Dental Clinic (Katowice, Poland), whereas Raman spectroscopic analyses were conducted at the Department of Biomedical and Dental Sciences and Morphofunctional Imaging, University of Messina (Italy). All samples were anonymized and used exclusively for in vitro research purposes; therefore, according to national regulations, ethical approval was not required.

### 2.2. Preparation and Storage of Extracted Teeth

Immediately after extraction, teeth were immersed in a 3% hydrogen peroxide solution for 3–5 min for initial decontamination. Specimens were mechanically cleaned under magnification (2.5–3.5×) to remove residual soft tissues, calculus, carious lesions, and restorative materials. Endodontically treated teeth were excluded.

Following cleaning, the specimens were re-immersed in 3% hydrogen peroxide for 5 min, dried using sterile gauze and compressed air, and sealed in sterilization pouches (ISO 11607-1:2019) [35]. Samples were labeled and stored at 4 °C until further processing.

No dehydration procedures were applied during storage in order to preserve dentin hydration and minimize alterations of the collagen–mineral interface [24,25].

### 2.3. Sample Retrieval and Fragmentation

Prior to processing, the teeth were removed from refrigerated storage and handled under standardized conditions. Samples were briefly immersed in 3% hydrogen peroxide (~3 min) for surface decontamination and subsequently dried using sterile gauze.

Each tooth was mechanically fragmented using a sterile mortar and surgical hammer to obtain dentin particles of heterogeneous size while preserving the native composite structure.

### 2.4. Preparation of Dentin-Derived Biomaterials

Three dentin-processing systems were used to generate biomaterial particles representing distinct preparation pathways.

For the BM system, dentin fragments were ground into particles (500–1000 µm) and subjected to an automated demineralization process using hydrochloric acid (3.5–5%), ethanol (70%), and hydrogen peroxide (5%) for 20 min 50 s.

For the TT system, fragments were milled (40–1200 µm) and processed using an automated cycle involving hydrogen peroxide, hydrochloric acid, and demineralized water with ultrasonic activation for 25 min.

For the SDG system, dentin was ground (300–1300 µm) and treated with sodium hydroxide (0.5 M) solution containing 20% ethanol, followed by neutralization in phosphate-buffered saline.

These protocols represent different approaches to modifying the collagen–mineral balance and structural organization of dentin-derived biomaterials [15,16,17].

Following processing, samples were fixed in 2.5% glutaraldehyde and transported under controlled conditions for spectroscopic analysis.

### 2.5. Raman Spectroscopy Analysis

BM, TT, SDG and Control teeth crushed particles were analyzed by using the Raman technique on a DXR-SmartRaman Spectrometer (Thermo Fisher Scientific, Waltham, MA, USA) equipped with a 785 nm excitation diode laser.

Prior to data acquisition, the instrument was calibrated using manufacturer-provided wavelength standards. Spectra were collected across a wavenumber range of 200 to 3200 cm^−1^ with a spectral resolution of approximately 2 cm^−1^. Irradiation was conducted using a laser power of 24 mW coming out through a 50 µm pinhole aperture. In order to obtain high signal-to-noise ratio (S/R) spectra, each Raman spectrum was obtained from 32 sample exposures; the length of each exposure during data collection was set to 30.0 s. Total acquisition time was 16 min for each spectrum. All the Raman spectra were stored in SPA format, and the post-processing analysis was performed using the Omnic for dispersive Raman 9.0 software.

To ensure data reliability, each sample was analyzed in triplicate. Prior to each measurement, the sample was repositioned to evaluate material homogeneity and to identify potential spatial variations in chemical composition.

In post-processing analysis, a manual baseline correction was applied to each Raman spectrum to compensate for eventual technical and/or sample variations, and then the mean spectrum of each acquired sample was obtained. Raman spectra were normalized using the vector normalization method previously employed by the authors in [29,30]. Specifically, each spectral intensity was divided by the Euclidean norm (the square root of the sum of the squared intensities of the spectrum), thereby standardizing the data for subsequent comparative analysis. In order to investigate the temporal stability of the organic-to-mineral ratio and identify any potential structural degradation mechanisms occurring over time inside the obtained spectra, we have calculated six different indices to characterize quantitatively and qualitatively the changes in organic and inorganic components. The crystallinity was evaluated based on the inverse of Full Width at Half Maximum (FWHM) of the phosphate band located at 960 cm^−1^ (Index 1 (I1)) [26]. In general, the narrower the spectral peak width is, the higher the degree of mineral crystallinity [36]. To obtain carbonate to phosphate (CPR) information, the ratio between the carbonate peak (1070 cm^−1^) and phosphate peak (960 cm^−1^) was obtained. The CPRratio index (I2) provides mineral quality of dentine [32]. The Relative Mineral Concentration (RMC ratio) index (I3) was calculated as the intensity ratio between the peaks located at 960 cm^−1^ and 1003 cm^−1^, respectively. This index indicates the maximum relative degree of mineralization, and it is crucial for the mechanical strength of dentine. The ratio controls how dentin resists force, responds to decay, and interacts with restorative materials [33]. The Mineral to Matrix Ratio (MMR), representing the proportion of inorganic mineral (hydroxyapatite) to the organic collagen matrix, was evaluated (I4) as the intensity ratio of peaks centered at 960 cm^−1^ and 1450 cm^−1^, respectively [26,33]. The collagen quality index (I5) was evaluated from the proline (Pro) to hydroxyproline (Hyp) ratio by using the intensity peaks located at 850 cm^−1^ and 870 cm^−1^, respectively [34,37]. The last index evaluated (I6) was the distribution of the organic components examined based on the ratio of intensity peaks centered at 2940 cm^−1^ and 2880 cm^−1^, respectively [38].

### 2.6. Statistical Analysis

Numerical data were expressed as mean, SD, median, minimum, and maximum. The Kolmogorov–Smirnov test was used to assess the normality tendency of the indices distributions. This revealed the existence of significant deviations from normality; therefore, nonparametric tests were used for statistical analysis. To compare the four groups (BM, TT, SDG, and Controls) in relation to the six indices, the Kruskal–Wallis test was applied. Depending on the statistical significance obtained, pairwise comparisons were made between the groups using Dunnett’s test [39]. For this analysis, Bonferroni correction was applied, whereby the overall alpha level was adjusted by dividing it by the possible number of pairwise comparisons that could be performed between the four groups. Therefore, the adjusted alpha level is 0.050/6 = 0.008 [40]. Some boxplots were created to better visualize the distribution of the indices for comparing four groups. The significance alpha level was set at 0.050. Statistical analyses were performed by using SPSS for Windows, version 26.0.

## 3. Results

### 3.1. Raman Spectroscopy Results

**Figure 2:**
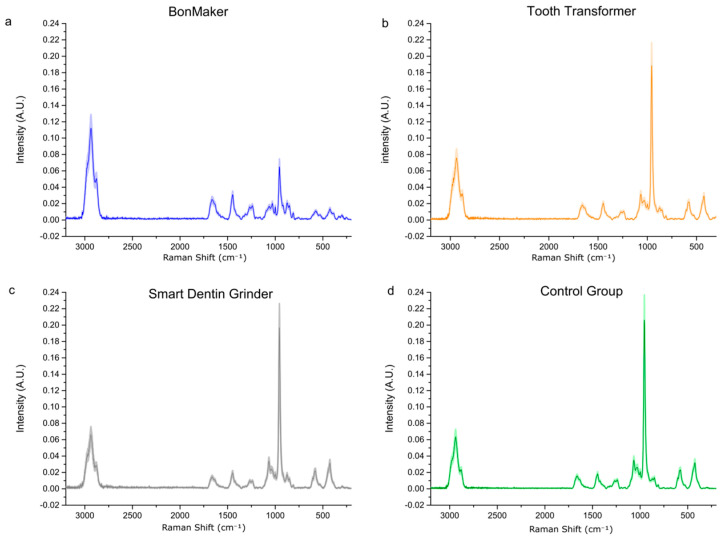
Raman spectra of the BM (**a**), TT (**b**), SDG (**c**), and control samples (**d**). Solid lines represent the mean spectrum, while the shaded area indicates the standard deviation.

The average Raman spectra of dentin-derived particles obtained from the BM, TT, SDG, and control groups are presented after baseline correction and vector normalization. Raman intensities are expressed in arbitrary units (a.u.) (Figure 2).

Overall, the Raman-derived indices demonstrated minimal temporal variation across all storage periods, whereas the observed variability was primarily associated with processing-dependent differences rather than storage duration.

The acquired spectra exhibited characteristic vibrational bands corresponding to both mineral and organic components of dentin. The principal Raman peaks and their biochemical assignments are summarized in Table 1.

The temporal stability of dentin as a hierarchical composite was evaluated using six Raman-derived indices describing mineral and organic components.

**Figure 3:**
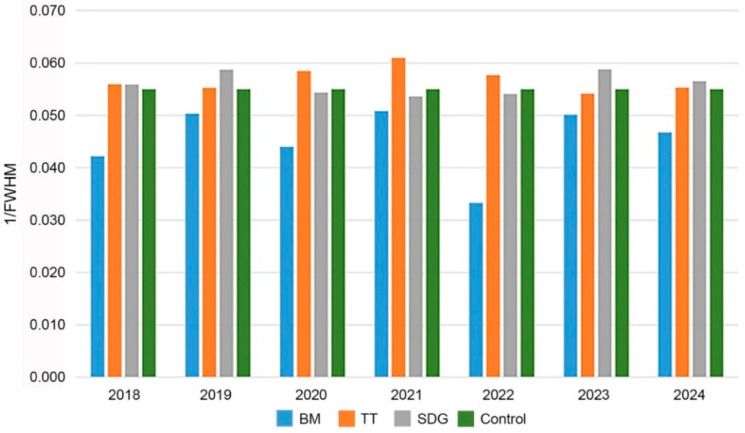
Mineral crystallinity (Index I1, 1/FWHM of the phosphate ν_1_ band at ~960 cm^−1^) evaluated across storage periods (2018–2024).

Mineral crystallinity (I1), expressed as the inverse FWHM of the phosphate ν_1_ band (~960 cm^−1^), demonstrated minimal variation across all storage groups between 2018 and 2024 (Figure 3). TT and SDG exhibited values closely resembling native dentin, whereas BM consistently showed lower crystallinity values compared with the other groups.

**Figure 4:**
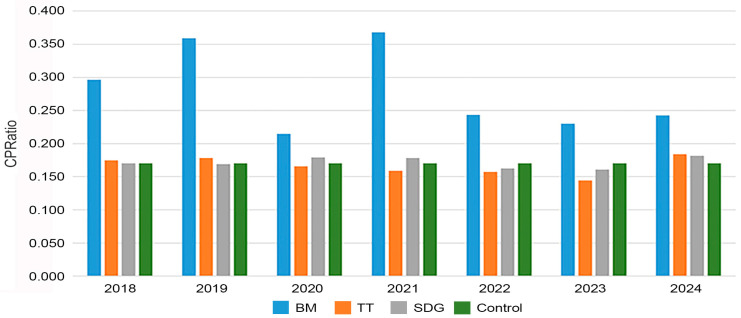
Carbonate-to-phosphate ratio (Index I2) evaluated across storage periods (2018–2024).

The carbonate-to-phosphate ratio (I2), reflecting mineral composition and maturity, remained stable across all storage periods (Figure 4). TT and SDG showed values comparable to the control group, while BM exhibited consistently higher carbonate substitution.

**Figure 5:**
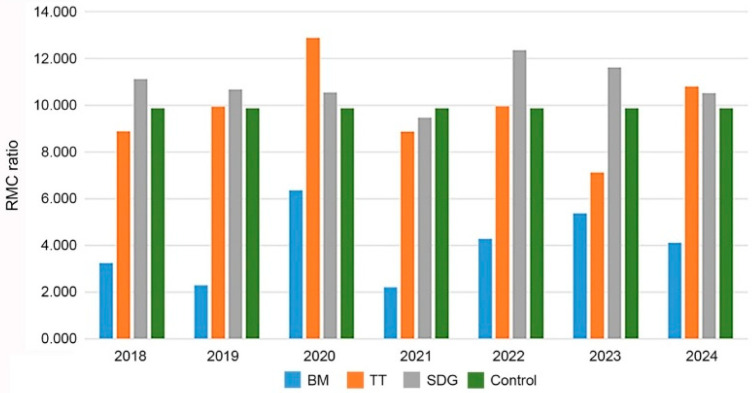
Relative mineral concentration (Index I3) evaluated across storage periods (2018–2024).

Analysis of the relative mineral concentration (I3) revealed more pronounced differences between processing groups (Figure 5). BM demonstrated lower mineral-related values compared with TT, SDG, and control samples, whereas TT and SDG remained closely aligned with native dentin throughout all storage periods.

**Figure 6:**
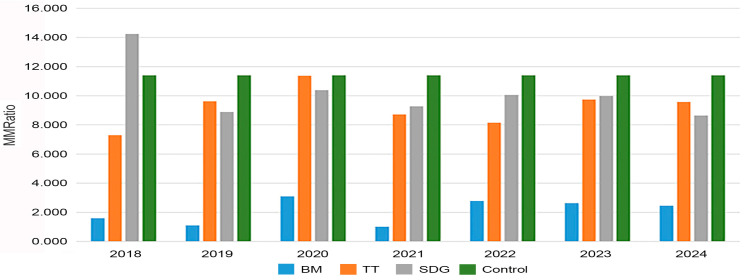
Mineral-to-matrix ratio (Index I4) evaluated across storage periods (2018–2024).

A similar pattern was observed for the mineral-to-matrix ratio (I4) (Figure 6). BM exhibited significantly lower values than the remaining groups, while TT and SDG maintained mineral-to-matrix ratios comparable to control dentin without evidence of temporal variation.

Notably, indices related to mineral content and mineral–matrix balance (I3 and I4) exhibited the most pronounced differences between groups, indicating that processing strategy was the dominant factor influencing the physicochemical characteristics of the biomaterials.

**Figure 7:**
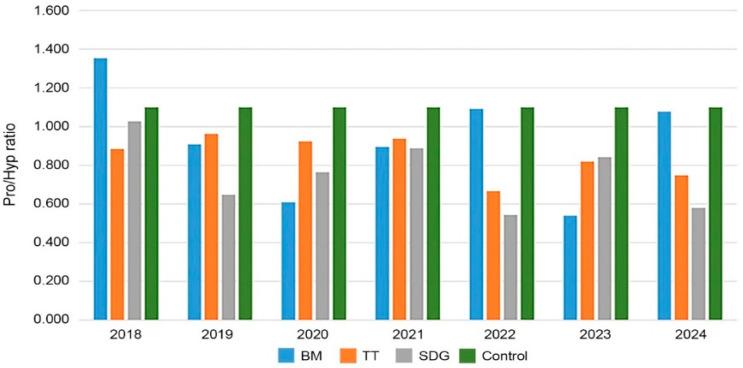
Collagen quality (Index I5; proline/hydroxyproline ratio) evaluated across storage periods (2018–2024).

The collagen quality index (I5), expressed as the proline-to-hydroxyproline ratio, showed only minor intergroup differences and no temporal trend (Figure 7). TT and SDG exhibited slightly lower values than the control group, whereas BM remained comparable to native dentin.

**Figure 8:**
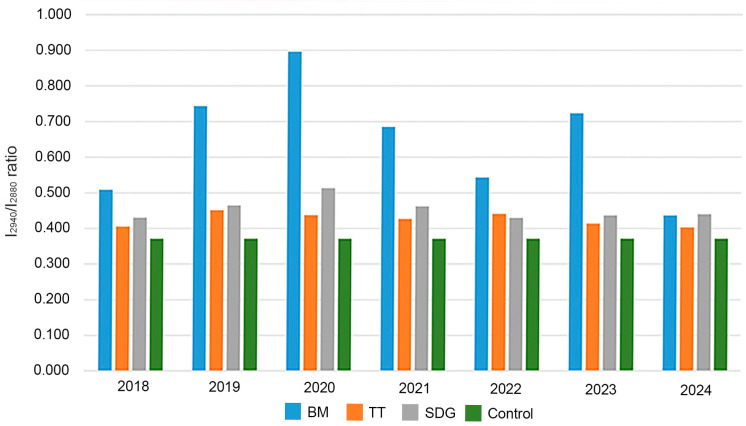
Organic matrix organization (Index I6; CH stretching ratio, 2940/2880 cm^−1^) evaluated across storage periods (2018–2024).

The organization of the organic matrix, assessed using the CH stretching ratio (I6), was higher in all processed groups compared with the control (Figure 8). BM exhibited the highest values, while TT and SDG also demonstrated elevated ratios relative to native dentin. Across all indices, no progressive changes associated with storage duration were observed. Overall, TT and SDG consistently exhibited Raman-derived profiles closely resembling native dentin, whereas BM demonstrated systematic deviations associated with its chemically modified processing pathway.

### 3.2. Statistical Analysis Results

Statistical analysis confirmed the trends observed in the Raman-derived indices, with significant differences primarily associated with processing-dependent variations rather than storage duration.

Pairwise comparisons among the four groups were performed for each index using Dunnett’s test with Bonferroni correction (Table 2). For indices I1 and I2, no statistically significant differences were observed after correction (*p* > 0.008), confirming comparable mineral crystallinity and carbonate substitution across groups.

In contrast, indices I3 and I4 showed highly significant differences between the BM group and all other groups (*p* ≤ 0.002), indicating reduced mineral content and altered mineral–matrix balance associated with this processing pathway. No significant differences were observed between TT, SDG, and control samples.

For the collagen-related index I5, no significant differences were detected between BM and the remaining groups. However, control samples exhibited significantly higher values compared with TT and SDG (*p* < 0.008).

For index I6, the control group demonstrated significantly lower values compared with all processed groups (*p* = 0.001), whereas no significant differences were observed between TT and SDG.

**Figure 9:**
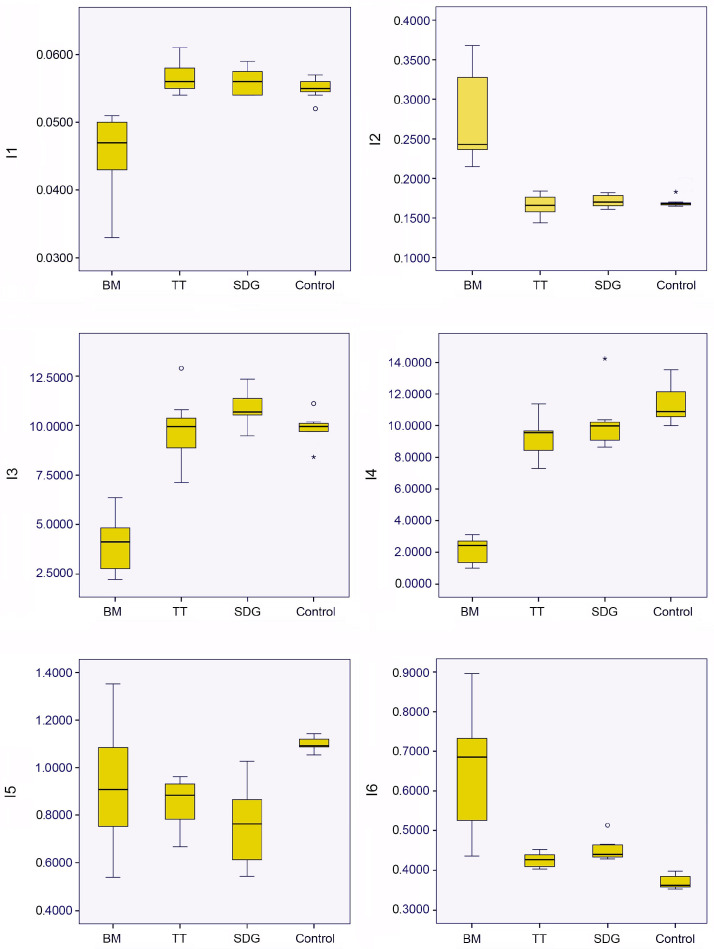
Distribution of Raman-derived indices (I1–I6) presented as box-and-whisker plots. The median is indicated by the central line, boxes represent the interquartile range (IQR), whiskers extend to 1.5× IQR. Circles (°) indicate outliers between 1.5× and 3× IQR from the box, whereas asterisks (*) indicate extreme outliers beyond 3× IQR.

The distribution of the Raman-derived indices is presented using box-and-whisker plots (Figure 9). The greatest intergroup separation was observed for indices associated with mineral content and mineral–matrix balance (I3 and I4), whereas indices related to mineral crystallinity and carbonate substitution (I1 and I2) demonstrated substantial overlap between groups.

Overall, the statistical analysis confirmed that the physicochemical stability of dentin-derived biomaterials was preserved during long-term refrigerated storage, while the observed differences were predominantly associated with processing-induced modifications rather than storage duration.

## 4. Discussion

The present study evaluated the physicochemical stability of dentin-derived biomaterials following prolonged refrigerated storage using Raman spectroscopy. The findings demonstrated that storage duration did not induce measurable time-dependent alterations in either mineral or organic components of dentin, whereas the observed differences were primarily associated with processing-dependent modifications.

While existing literature has extensively characterized the short-term physicochemical properties of dentin-derived scaffolds immediately following their preparation, the long-term stability of these hierarchical collagen–mineral composites during prolonged refrigerated storage remains a subject of limited investigation. To our knowledge, this study addresses a critical knowledge gap by evaluating the physicochemical integrity of dentin-derived biomaterials after up to six years of storage. By providing longitudinal empirical evidence, these findings offer essential insights into the stability of these scaffolds, thereby substantiating the practical feasibility and clinical safety of tooth banking protocols for bone regenerative applications.

Dentin is a hierarchically organized collagen–mineral composite in which structural integrity is governed by the interaction between type I collagen fibrils and carbonated hydroxyapatite nanocrystals [1,2,3,19]. This hierarchical organization is responsible for the mechanical stability and physicochemical behavior of mineralized tissues [1,4,5]. Disruption of the collagen–mineral interface may alter mineral crystallinity, collagen organization, and interfacial interactions, ultimately affecting the functional properties of the material.

Although complementary techniques such as SEM, XRD, or mechanical testing would provide deeper insights into the ultrastructural morphology and mechanical performance, Raman spectroscopy was used as our primary tool because it allows for the simultaneous, non-destructive, and label-free analysis of both the mineral and organic (collagen) components of dentin in its native-like state. While techniques such as SEM, FTIR, and mechanical testing would provide excellent complementary data, they often require sample dehydration or preparation protocols that could induce artifacts, potentially altering the very collagen–mineral interface we aimed to observe in its “stored” state.

In the present study, Raman-derived indices associated with mineral structure, including mineral crystallinity (I1) and carbonate substitution (I2), remained stable across all storage periods. These findings suggest preservation of the mineral phase during prolonged refrigerated storage and are consistent with previous reports describing the structural stability of apatite under controlled environmental conditions [2,6].

Similarly, indices related to the organic matrix, including collagen quality (I5) and matrix organization (I6), did not demonstrate progressive temporal changes. The stability of collagen-rich mineralized tissues depends strongly on hydration-mediated interactions and intermolecular collagen organization [9,10,23,24,25]. Preservation of collagen-associated Raman signatures throughout all storage periods therefore suggests maintenance of the collagen–mineral interface and its hydration-dependent structural integrity.

An important finding of this study is the distinction between storage-related stability and processing-induced modification. The results consistently demonstrated that processing strategy, rather than storage duration, represented the dominant factor influencing the physicochemical characteristics of dentin-derived biomaterials. In particular, indices associated with mineral content and mineral–matrix balance (I3 and I4) exhibited the greatest intergroup differences.

The BM system demonstrated reduced mineral-related indices together with an increased relative contribution of the organic matrix, reflecting the effects of its chemically driven demineralization protocol. These observations are consistent with previous studies reporting that acid-based dentin processing modifies the mineral–organic balance and alters the structural characteristics of dentin-derived graft materials [15,16,17].

In contrast, TT and SDG exhibited Raman-derived profiles closely resembling native dentin, suggesting preservation of the intrinsic collagen–mineral architecture. These findings support the concept that minimally modified or mineral-preserving processing approaches maintain the hierarchical organization of dentin-derived biomaterials more effectively than extensive demineralization procedures [16,17,18].

From a methodological perspective, Raman spectroscopy proved to be a suitable tool for evaluating the physicochemical stability of dentin-derived biomaterials. Its ability to simultaneously characterize mineral and organic components enables indirect assessment of the collagen–mineral interface, which represents a critical determinant of the structural behavior of mineralized tissues [26,27,31,32,33,34]. Although Raman-derived indices do not constitute direct ultrastructural measurements, they provide sensitive and reproducible descriptors for comparative analysis of hierarchical biomaterials.

The present findings may also have translational relevance for regenerative dentistry and future tooth banking strategies. The demonstrated stability of dentin during prolonged refrigerated storage suggests that extracted teeth may retain their physicochemical properties prior to biomaterial processing, potentially facilitating delayed clinical use and improving flexibility in regenerative workflows. However, although spectroscopic stability was preserved, further studies are required to determine whether long-term storage may influence osteoinductive potential, biological remodeling, or in vivo regenerative performance.

Several limitations should be considered when interpreting the present results. The study focused primarily on spectroscopic characterization and did not directly evaluate mechanical properties or biological response. In addition, subtle nanoscale alterations not detectable by Raman spectroscopy cannot be completely excluded. Future investigations integrating spectroscopic, ultrastructural, mechanical, and biological analyses are therefore warranted to provide a more comprehensive characterization of stored dentin-derived biomaterials.

Overall, the findings indicate that dentin exhibits substantial physicochemical stability as a hierarchical biomaterial during long-term refrigerated storage, while the final characteristics of dentin-derived scaffolds are predominantly determined by processing-dependent modifications of the collagen–mineral balance.

## 5. Limitations

The present study focuses on the molecular-level physicochemical characterization of dentin-derived biomaterials and does not directly assess their mechanical performance or biological response. Although Raman spectroscopy provides detailed insight into the structural organization of the collagen–mineral interface, complementary techniques such as nanoindentation, micro-mechanical testing, or cell-based assays would be required to establish direct structure–function relationships.

In addition, the relatively limited number of specimens per subgroup may limit statistical power to detect subtle differences between experimental conditions. However, the consistency of Raman-derived indices across time points supports the robustness of the observed trends.

Another potential limitation of this study is the use of 2.5% glutaraldehyde for sample preparation. While this fixative is known to induce collagen cross-linking, its application was standardized across all experimental groups and storage periods. Given that the Raman indices (such as I5) are calculated as relative ratios of peak intensities, the systematic use of this fixation protocol ensures that any potential spectral shift is uniform across all samples. Consequently, the observed longitudinal stability is a robust finding, reflecting the preserved integrity of the dentin scaffold despite the fixation treatment.

Furthermore, we know that multi-scale, multi-modal characterization, including Scanning Electron Microscopy (SEM) and X-ray Diffraction (XRD), represents the gold standard for full ultrastructural and crystallographic validation of hierarchical biomaterials. Evaluating the complete matrix of experimental conditions—encompassing three processing systems and multiple storage annual groups against freshly extracted controls—via SEM would yield an extensive volume of morphological data. Incorporating such a vast dataset would significantly exceed the balanced scope and structural limits of the present manuscript. Therefore, as a crucial development of this research line, a comprehensive multi-modal study focused exclusively on the ultrastructural visualization of fibrillar arrangement via SEM and crystallite size determination via XRD across the entire longitudinal cohort will be dedicated to a forthcoming standalone study.

In addition, the in vitro design of the study may limit the direct translation of the findings to clinical conditions, where biological remodeling processes may influence material behavior over time. While no measurable changes were observed in the Raman-derived indices, subtle nanoscale or ultrastructural alterations cannot be completely excluded. Future studies integrating spectroscopic, mechanical, and biological analyses are therefore warranted to provide a more comprehensive evaluation of dentin-derived biomaterials.

## 6. Conclusions

Within the limitations of this study, our findings indicate that dentin-derived biomaterials maintain a high degree of physicochemical stability as hierarchical collagen–mineral composites, even after prolonged refrigerated storage of up to six years. Time-resolved Raman spectroscopic analysis revealed that mineral crystallinity, carbonate substitution, mineral-to-matrix balance, and collagen-associated vibrational signatures remained stable over the observation period, suggesting that the molecular integrity of the collagen–mineral interface is preserved under these specific storage conditions. It is important to note that these conclusions are based on the sensitivity and scope of the Raman indices analyzed. While our data suggest that the structural integrity as probed at the molecular level is not significantly altered by storage duration, we acknowledge that this molecular stability does not fully account for macro-mechanical or long-term biological performance. The observed physicochemical characteristics of the scaffolds appear to be primarily governed by the initial processing methods rather than the length of pre-processing storage. From a biomaterials perspective, these results provide a promising molecular baseline supporting the use of dentin as a robust, bio-derived scaffold. While this observed stability may facilitate more flexible clinical workflows and support the potential for tooth banking approaches, these molecular indicators should be considered preliminary. Further longitudinal studies, incorporating multi-modal assessments such as mechanical testing and biological validation, are strictly required to confirm the long-term clinical relevance and functional performance of these materials.

## Figures and Tables

**Table 1:** Assignment of characteristic Raman peaks of dentin and corresponding biochemical components.

Raman Shift (cm^−1^)	Assignment	Biochemical Component	Reference
~431	ν_2_ PO_4_^3−^ bending	Phosphate (apatite)	[41]
~591	ν_4_ PO_4_^3−^ bending	Phosphate (apatite)	[41]
~850	Proline	Collagen	[41,42]
~870	Hydroxyproline	Collagen	[41,42]
~960	ν_1_ PO_4_^3−^ symmetric stretching	Phosphate (apatite)	[41]
~1003	Phenylalanine ring breathing	Protein (collagen)	[43]
~1065	CO_3_^2−^ substitution/C–O stretching	Carbonate (apatite)	[44]
~1265	Amide III	Collagen	[41,42]
~1450	CH_2_ deformation	Organic matrix	[41]
~1660	Amide I	Collagen (secondary structure)	[41]
~2880–2940	CH stretching	Organic matrix	[41,42]

Abbreviations: PO_4_^3−^—phosphate; CO_3_^2−^—carbonate.

**Table 2:** Pairwise comparisons among experimental groups for Raman indices I1–I6 (Dunnett’s post hoc test with Bonferroni correction). Significant values (*p* < 0.008) are indicated in bold.

Comparison	I1	I2	I3	I4	I5	I6
BM vs. TT	0.013	0.014	**0.002**	**0.001**	0.979	0.048
BM vs. SDG	0.018	0.019	**0.001**	**0.001**	0.701	0.084
BM vs. Control	0.031	0.018	**0.001**	**0.001**	0.542	0.018
TT vs. SDG	0.991	0.929	0.621	0.808	0.793	0.273
TT vs. Control	0.569	0.988	0.998	0.060	**0.003**	**0.001**
SDG vs. Control	0.905	0.995	0.215	0.703	**0.007**	**0.001**

Abbreviations: BM, BonMaker; TT, Tooth Transformer; SDG, Smart Dentin Grinder.

## Data Availability

The data supporting the findings of this study are available from the corresponding author upon reasonable request.
